# Omega-6 and Omega-3 Fatty Acid-Derived Oxylipins from the Lipoxygenase Pathway in Maternal and Umbilical Cord Plasma at Delivery and Their Relationship with Infant Growth

**DOI:** 10.3390/ijms23020708

**Published:** 2022-01-09

**Authors:** Maranda Thompson, Arzu Ulu, Ana G. Yuil-Valdes, Maheswari Mukherjee, Melissa Thoene, Matthew Van Ormer, Rebecca Slotkowski, Elizabeth Lyden, Ann Anderson Berry, Corrine K. Hanson, Tara M. Nordgren, Sathish Kumar Natarajan

**Affiliations:** 1 Pediatrics Department, University of Nebraska Medical Center, Omaha, NE 68198, USA; maranda.thompson@unmc.edu (M.T.); melissak.thoene@unmc.edu (M.T.); matthew.vanormer@unmc.edu (M.V.O.); rebecca.slotkowski@unmc.edu (R.S.); alanders@unmc.edu (A.A.B.); 2 Division of Biomedical Sciences, University of California Riverside, Riverside, CA 92521, USA; arzu.ulu@medsch.ucr.edu (A.U.); tara.nordgren@colostate.edu (T.M.N.); 3 Department of Pathology and Microbiology, University of Nebraska Medical Center, Omaha, NE 68198, USA; ana.yuilvaldes@unmc.edu; 4 Cytotechnology Education, College of Allied Health Professions, University of Nebraska Medical Center, Omaha, NE 68198, USA; mmukherj@unmc.edu; 5 Department of Biostatistics, College of Public Health, University of Nebraska Medical Center, Omaha, NE 68198, USA; elyden@unmc.edu; 6 Medical Nutrition Education, College of Allied Health Profession, University of Nebraska Medical Center, Omaha, NE 68198, USA; ckhanson@unmc.edu; 7 Department of Environmental and Radiological Health Science, Colorado State University, Fort Collins, CO 80525, USA; 8 Department of Nutrition & Health Sciences, University of Nebraska-Lincoln, Lincoln, NE 68583, USA

**Keywords:** omega-3 fatty acids, omega-6 fatty acids, metabolites, oxylipins, plasma, delivery, infant growth, inflammation, lipoxygenase, birth weight percentile, birth length percentile, birth head circumference percentile

## Abstract

Omega-3 and omega-6 fatty acids are important for neonatal development and health. One mechanism by which omega-3 and omega-6 fatty acids exert their effects is through their metabolism into oxylipins and specialized pro-resolving mediators. However, the influence of oxylipins on fetal growth is not well understood. Therefore, the objective of this study was to identify oxylipins present in maternal and umbilical cord plasma and investigate their relationship with infant growth. Liquid chromatography–tandem mass spectrometry was used to quantify oxylipin levels in plasma collected at the time of delivery. Spearman’s correlations highlighted significant correlations between metabolite levels and infant growth. They were then adjusted for maternal obesity (normal body mass index (BMI: ≤30 kg/m^2^) vs. obese BMI (>30 kg/m^2^) and smoking status (never vs. current/former smoker) using linear regression modeling. A *p*-value < 0.05 was considered statistically significant. Our study demonstrated a diverse panel of oxylipins from the lipoxygenase pathway present at the time of delivery. In addition, both omega-3 and omega-6 oxylipins demonstrated potential influences on the birth length and weight percentiles. The oxylipins present during pregnancy may influence fetal growth and development, suggesting potential metabolites to be used as biomarkers for infant outcomes.

## 1. Introduction

Pregnancy is a period of rapid fetal growth and cell differentiation in the womb, where diverse insults lead to acute diseases with untoward long-term consequences. The fetus is susceptible to inflammatory stimulation that may affect embryonic growth, placental development, organogenesis, and regulation of immune responses [1]. An inflammatory uterine environment can increase the risk of premature infants developing various diseases at later life stages. Examples include chronic lung diseases, retinopathy of prematurity, intraventricular hemorrhage, periventricular leukomalacia, and necrotizing enterocolitis [2,3]. These considerations underscore the importance of identifying modifiable factors that can reduce and limit the negative consequences of inflammation in the intrauterine environment.

Maternal nutrition is a modifiable factor that impacts infant outcomes [4,5]; for example, fatty acids (FAs) are essential for proper pregnancy progression and normal fetal growth [6,7]. Omega (n)-3 FAs are necessary for fetal brain and eye development [1]. In addition, dietary n-3 supplementation in pregnancy has been shown to reduce preterm births [8,9,10,11,12], intrauterine growth restriction [6], and admission to the neonatal intensive care unit [13,14]. A phase III randomized controlled trial in which a cohort of women received supplemental n-3 FAs during pregnancy revealed that this intervention increased infant birth weight, length, and head circumference [15]. Omega n-6 FAs are essential nutrients that become incorporated into the phospholipid bilayer. Although their effects are mixed, these lipids play vital roles in organ development and function [1]. The Western diet is high in n-6 FAs and relatively low in n-3 FAs; notably, pregnant women have reported lower intakes of n-3 FAs compared to nonpregnant women [14,16]. Excess n-6 FA consumption has been associated with increased anxiety in animal models [1], the early-life onset of obesity and metabolic diseases [17], and fetal cardiovascular health [18]. Mechanistically, excess maternal intake of n-6 FAs can limit the metabolism of beneficial n-3 FAs as all FAs compete for the same metabolic enzymes. This competition impacts the balance of n-3 and n-6 FAs available to the fetus and may adversely affect fetal growth and pregnancy outcomes [19].

Accumulating evidence suggests that n-6 and n-3 FAs affect maternal and fetal health through their metabolism into biologically active eicosanoids. The generation of these signaling molecules is catalyzed by lipoxygenase (LOX), cyclooxygenase (COX), and cytochrome-P-450 (CYP450)-dependent oxygenases. Omega-6 FA metabolism generates pro-inflammatory eicosanoids such as lipoxins and leukotrienes. In contrast, n-3 FAs are precursors of anti-inflammatory eicosanoids and specialized pro-resolving mediators (SPMs) that exhibit protective functions [20,21,22]. Eicosanoids and their bioactive metabolites play central roles at all stages of inflammation, including initiation, progression, and resolution.

While a large body of work has advanced our understanding of the biological role of eicosanoids, the impact of the LOX pathway and its metabolites in pregnancy remains to be established. Mozurkewich et al. demonstrated that n-3 supplementation during pregnancy augments LOX pathway metabolites [23]. However, most reports are limited to analyses of metabolite levels up to 35 weeks of gestation, do not analyze potential relationships between metabolite levels in maternal and umbilical cord plasma, or investigate their impact on birth outcomes. This study characterized LOX pathway metabolites in maternal and umbilical cord plasma at the time of delivery and explored the relationship between LOX metabolite levels and birth outcomes.

## 2. Results

### 2.1. Baseline Characteristics

#### 2.1.1. Maternal Demographics

One hundred twenty-one mother–infant pairs were available for analysis. The mean maternal age assessed in 118 women was 29.36 years (SD = 5.88). Table 1 and Table 2 summarize maternal characteristics within our cohort. Twenty-eight percent of mothers had an obese pre-pregnancy BMI (BMI > 30 kg/m^2^), and 22% were former or current smokers at the time of delivery. Vaginal deliveries were performed for 74.58% of births and 25.42% via Caesarean Section (CS).

The median maternal daily dietary intake of total n-6 and n-3 fatty acids was 14.46 g/day [7.432] and 1.72 g/day [1.01], respectively. Total n-3 fatty acid intake includes fish oil supplementation, ALA, EPA, and DHA. However, ALA is minimally converted into EPA and DHA. Twenty percent of mothers reported fish oil supplementation during pregnancy.

#### 2.1.2. Infant Demographics

Infants were, on average, born at 38.2 weeks (3.29) of gestation (Table 3). They weighed 3.2 kg at birth and measured at 34 cm for head circumference and 49 cm for birth length. Infants were in the ~50th percentile for birth weight, length, and head circumference. There was no significant difference between the number of male vs. female infants included in this study.

#### 2.1.3. Association between Metabolite Levels and Baseline Characteristics

Cord plasma n-6 metabolite levels differed significantly when delivery modes were compared. Umbilical cord samples from CS vs. vaginal deliveries had lower median levels of 9-HODE (7.26 nM vs. 10.52 nM; *p* = 0.010), 9-HOTrE (0.35 nM vs. 0.52 nM; *p* = 0.003), and 13-KODE (1.05 nM vs. 1.52 nM, *p* = 0.039). Further, umbilical cord plasma 5,15-DiHETE was higher in CS compared to vaginal deliveries (median: 0.24 nM vs. 0.14 nM; *p* = 0.036). There were no significant differences in plasma metabolite levels and infant sex.

To unveil potential relationships between eicosanoid levels and outcomes, LTB4, 9-HEPE, and 8,15-DiHETE were classified as detectable and nondetectable. There was a significant difference in AA intake by subjects with detectable vs. nondetectable maternal LTB4 levels. A maternal intake of AA around 0.29 g/day was associated with detectable LTB4 in maternal plasma. Further, there was a relationship between maternal LTB4 levels and birth weight percentile. The mean birth weight percentile was 59.71 % (*n* = 103) in the nondetectable LTB4 group compared with 37.72% (*n* = 13) in the detectable LTB4 cohort. There appeared to be a relationship between BMI and the ability to detect LTB4 levels. Higher BMI status was associated with few samples with detectable LTB4 levels.

### 2.2. Relationships between LOX Metabolite Levels in Maternal and Umbilical Cord Plasma

#### 2.2.1. Correlations for Parent Nutrients and n-6 Metabolites in Maternal and Umbilical Cord Plasma

The relationships between omega-6 parent FAs and metabolites were explored (Table 4). As maternal AA levels increased, cord 15-HETE and lipoxin A4 were negatively correlated. Appendix A illustrates the median concentrations of the parent FAs analyzed in this study.

To understand the presence of n-6 oxylipins at delivery, we explored the correlations between maternal and cord plasma. We observed significant correlations in the levels of n-6 PUFA metabolites in maternal and cord plasma. AA and DGLA’s metabolites were higher in cord than maternal plasma, whereas LA precursors tended to be lower in cord vs. maternal specimens (Table 5).

#### 2.2.2. Correlations for Parent Nutrients and n-3 Metabolites in Maternal and Umbilical Cord Plasma

Table 6 illustrates the correlations between maternal parent omega-3 FA nutrients with omega-3 metabolites in maternal and umbilical cord blood. We found that maternal EPA in plasma had a significant, negative correlation with maternal 17-HDHA. The same relationship was found between maternal EPA levels and cord 17-HDHA. Maternal parent nutrient concentrations are displayed in Appendix B.

We identified EPA and DHA metabolites and found that these lipids tended to be higher in maternal than cord plasma. Table 7 shows maternal and cord metabolite levels and displays the significant, positive correlations between maternal and infant metabolites. ALA metabolite 9-HOTrE was not significantly correlated between maternal and cord plasma.

### 2.3. Metabolite Association with Maternal Diet

The influence of maternal dietary intake was analyzed to understand the impact of diet on metabolite levels in maternal and cord plasma. Total n-6 PUFA intake was positively correlated with cord 15-HETE levels. In contrast, the maternal intake ratio of n-6 to n-3 was negatively correlated with maternal 15-HETE (AA metabolite) and 17-HDHA (DHA metabolite). When analyzing the influence of DHA intake on metabolite levels, we found that DHA intake was significantly positively correlated with maternal 9-HEPE, 7-HDHA, and 17-HDHA plasma levels. Total omega-3 fatty acid intake, however, was only significantly positively correlated with cord 17-HDHA. Significantly lower median n-6 metabolite concentrations were observed in individuals who used fish oil supplements, including cord 15-HETrE (median: 1.05 nM vs. 1.50 nM, *p* = 0.032) and maternal 5,15-DiHETE (median: 0.08 nM vs. 0.28 nM, *p* = 0.027), both n-6 metabolites. Table 8 shows significant correlations between dietary intakes and metabolite plasma levels.

### 2.4. Associations between Fatty Acid Metabolites and Infant Growth

#### 2.4.1. The Relationships between n-6 FA Metabolites and Infant Growth

**Figure 1:**
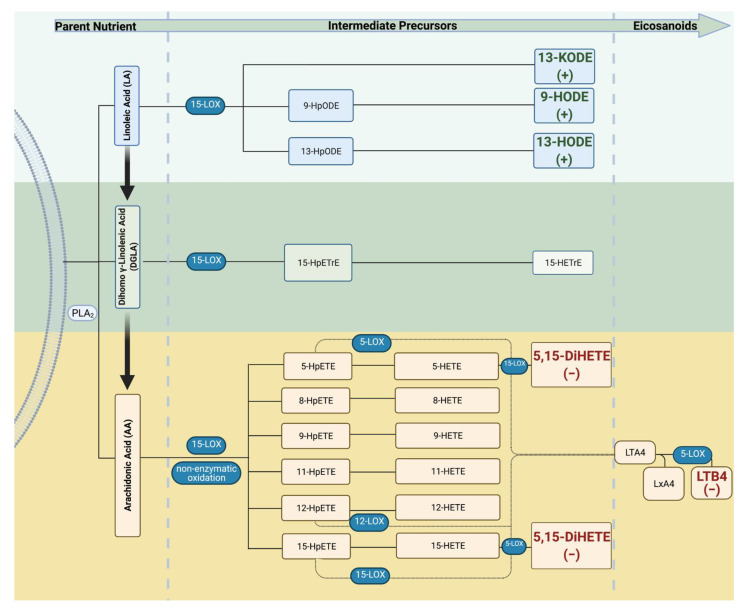
Lipoxygenase (LOX) enzymatic breakdown of n-6 PUFA. Omega-6 FAs are enzymatically cleaved by phospholipase A_2_ (PLA_2_). Parent n-6 FA can then interact with LOX enzymes to be further metabolized into oxylipins that participate in the physiological process. Increases in cord LA metabolites, *13-KODE, 13-HODE***,** and *9-HODE***,** predicted an increase in birth length percentile. In contrast, higher levels of *maternal 5,15-DiHETE* were associated with a decrease in birth weight percentile. Created with BioRender.com (30 December 2021). (+), beta value was positive; (−), beta value was negative; HpODE, hydroxyperoxyoctadecadienoic acid; KODE, ketooctadecadienoic acid; HODE, hydroxyoctadecadienoic acid; HpETrE, hydroxyperoxyeicosatrienoic acid; HETrE, hydroxyeicosatrienoic acid; HpETE, hydroxyperoxyeicosatetraenoic acid; HETE, hydroxyeicosatetraenoic acid; DiHETE, dihydroxyeicosatetraenoic acid; LT, leukotriene; LX, lipoxin.

The influence of n-6 FA metabolites on infant growth was analyzed using Spearman correlations (Table 9). Significant relationships were then adjusted for maternal smoking status and pre-pregnancy BMI. Cord 9-HODE, 13-HODE, and 13-KODE predicted birth length percentile. With every 1% increase in cord 9-HODE, 13-HODE, and 13-KODE, infant birth length percentile increased by 0.13, 0.20, and 0.025, respectively. However, the infant birth weight percentile decreased by 0.041 for every 1% increase in maternal 5,15-DiHETE. Figure 1 illustrates the relationship between the significant metabolites in the LOX pathway.

#### 2.4.2. n-3 FA Metabolites and Infant Growth

**Figure 2:**
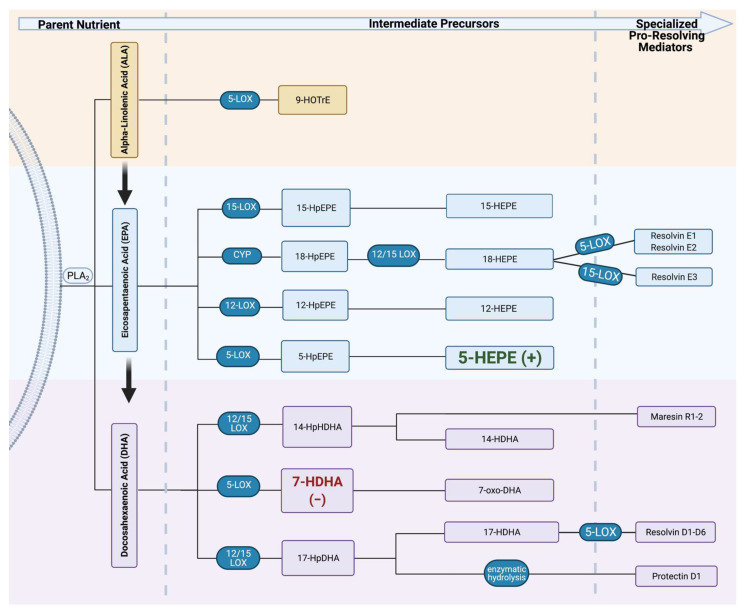
Lipoxygenase (LOX) enzymatic breakdown of n-3 PUFA. Omega-3 FAs are enzymatically cleaved by phospholipase A_2_ (PLA_2_) from the phospholipid bilayer. Parent n-3 FA can then interact with the same LOX enzymes that break down n-6 to be metabolized into oxylipins with less potent anti-inflammatory properties. Increases in *cord 5-HEPE* were predictive of an increase in birth length percentile. However, increases in *maternal 7-HDHA* were predictive of a decrease in birth weight percentile. Created with BioRender.com (30 December 2021). (+), beta value was positive; (−), beta value was negative; HOTrE, hydroxyoctadecatrenoic acid; HpEPE, hydroxyeicosatetraenoic acid; HEPE, hydroxyeicosapentaenoic acid; HpHDHA, hydroxyperoxydocosahexaenoic acid; HDHA, hydroxydocosahexaenoic acid; oxo-DHA, oxo-docosahexaenoic acid.

We assessed potential relationships between infant growth and n-3 FA metabolites using Spearman correlations (Table 10). We found that the n-3 metabolites 5-HEPE in cord and maternal specimens, respectively, were associated with infant growth after adjustment for maternal smoking and pre-pregnancy BMI status. For every 1% increase in median cord 5-HEPE (EPA metabolite), the birth length percentile increased by about 0.12. In contrast, the expected birth weight percentile decreased by 0.062 for every 1% increase in maternal 7-HDHA. Figure 2 highlights the significant metabolites in the LOX pathway. No other significant relationships between n-3 metabolites and infant growth were identified.

## 3. Discussion

The main findings of our study highlight the relationship between LOX pathway metabolites in maternal and cord plasma and the impact these metabolites have on infant growth metrics. Metabolites 5-HEPE, 9-HODE, 13-HODE, and 13-KODE positively predicted infant birth anthropometrics, while 7-HDHA and 5,15-DiHETE negatively predicted infant growth. Our study is the first to explore the relationship between n-6 and n-3 LOX pathway metabolite exposure in utero and fetal growth outcomes at the time of delivery.

### 3.1. n-6 PUFA

AA metabolites play essential roles in the continued maintenance of pregnancy and delivery. Interestingly, we identified a trend toward higher circulating AA metabolite levels in cord plasma compared to maternal cord plasma. In addition, cord 15-HETE and lipoxin A4 were negatively associated with AA in maternal plasma. Previous studies reported decreased parent nutrient AA in maternal plasma accompanied by increases in cord plasma throughout pregnancy. Best et al. identified a similar trend in AA metabolites; this group reported lower 8-, 9-, 11-, 12-, and 15-HETE levels in maternal blood spots at 34 vs. 14 weeks of gestation. In contrast, maternal 5-HETE levels increase significantly throughout pregnancy [24] and have been shown to induce uterine contractility [25]. HETEs have essential functions: they function as PPAR ligands due to their binding capacity to nuclear receptor transcription factors. Additionally, they participate in protein kinase signaling activation, angiogenesis, and neuronal apoptosis [21]. Further, the ability of 12- and 15-HETE to activate the capsaicin-sensitive receptor correlates with pain signaling during inflammation [21]. We found similar increases in AA metabolites in cord vs. maternal plasma at the time of delivery. Arachidonic acid concentrations, the precursor for HETE metabolites, are much higher in fetal blood and tissues compared to maternal blood. However, there is little evidence that placental chain elongation and desaturation account for the observed increases in AA supplied to the fetus [26].

The impact of AA metabolites on pregnancy outcomes remains to be precisely defined. Both beneficial and adverse effects have been reported. Most studies investigated potential relationships between metabolite levels in maternal plasma collected before delivery and maternal/infant outcomes. Goveia-Figueira et al. found that 5- and 15-HETE levels were associated with premature labor [27]. Similarly, Ramsden et al. showed that above-median 5-HETE and 15-HETE concentrations were associated with a higher risk of preterm delivery when assessed at gestational week 14 [28]. Maternal plasma samples from women with pre-eclampsia had significantly higher 5-, 8-, 12-, 15-HETE, and LTB4 levels compared with women having normal pregnancies and nonpregnant women. Moreover, 5-HETE, 15-HETE, and LTB4 levels were significantly higher in plasma from women with severe vs. mild pre-eclampsia.

Few studies have addressed the importance of LOX metabolites in maternal and cord plasma on infant growth. Welch et al. used Bayesian modeling to demonstrate an association between the adjusted mean concentration of 12-HETE and size for gestational age. Among gestational age cases who were small, the adjusted mean concentration of maternal 12-HETE was 56.2% higher than that observed in mothers whose infants were classified as appropriate for gestational age [29]. We found no predictive value for maternal or cord 12-HETE on infant growth metrics. Still, we observed that maternal plasma levels of the AA metabolite 5,15-DiHETE were negatively associated with infant birth weight percentile at birth.

The impact of cord plasma AA metabolites on infant outcomes is not well understood. A previous study found that in pre-eclamptic pregnancies, umbilical cord constriction is dependent on 15-HETE levels [30]. In our research, cord AA metabolites were not significantly associated with infant growth, but we observed a trend toward lower LA metabolite levels in cord vs. maternal plasma. LA is an essential n-6 fatty acid metabolized into HODEs through the LOX pathway. HODEs, as with HETEs, are generally present in healthy pregnancies [21]; they function as PPAR ligands and have atherogenic properties. HODE activity is modulated by Th1 and Th2 lymphocytes with cytokine and chemokine participation, either pro- or anti-inflammatory, depending on the type of lymphocytes present [31]. Best et al. demonstrated that maternal 13-HODE significantly decreases throughout normal pregnancy, suggesting that less metabolite is transferred to the developing fetus [24]. On the other hand, pregnancies with maternal 9-HODE levels below the median concentration tended to have a higher risk of spontaneous preterm birth [28]. Our results show that cord 13-HODE, 9-HODE, and 13-KODE are positively associated with birth length percentile. Together, our experimental results lead us to propose that AA and LA metabolites likely regulate events necessary for normal delivery and potentially influence fetal growth and blood vessel development through their role as PPAR ligands. In addition, elevated HETEs appear to be associated with pregnancy pathologies such as pre-eclampsia, impaired infant growth, and preterm delivery.

### 3.2. n-3 PUFA

EPA and DHA are n-3 PUFAs that compete with n-6 fatty acids for LOX pathway enzymes to produce a class of less potent anti-inflammatory specialized pro-resolving mediators (SPMs) [32]. EPA is converted to HEPEs and further metabolized into the SPM Resolvin E-series. HEPEs induce neutrophil chemotaxis, inhibit platelet aggregation, and play a role in mouse adipogenesis [33]. The resolvin E-series inhibit the further inflammation and release of cytokines and are involved in the resolution of inflammatory responses. A previous study reported higher EPA, SPMs, and metabolites in infant cord blood after maternal dietary n-3 supplementation [24]. However, these studies did not evaluate the relationship between metabolite levels and infant outcomes. In our research, EPA 5-HEPE levels in maternal and cord plasma were significantly correlated, but maternal plasma levels of EPA were not significantly correlated with EPA metabolite levels. Further, 5-HEPE positively predicted infant length percentile at birth. It remains to be established whether these correlations reflect the active participation of 5-HEPE in fetal outcomes. Mechanistic analyses have shown that 5-HEPE enhances glucose-dependent insulin secretion in mouse insulinoma cells and human intestinal carcinoma cells [33,34]. It is tempting to hypothesize that 5-HEPE is a fetal growth factor that regulates infant growth through its effects on insulin secretion.

Many DHA-derived resolvins are synthesized through metabolic steps catalyzed by 15-LOX and 5-LOX. 17-HDHA is a 15-LOX intermediate and a pathway marker for the D-series resolvins. Mozurkewich et al. demonstrated that umbilical cord 17-HDHA was significantly increased compared with maternal levels at 12–20 and 34–36 weeks of gestation [23]. In these investigations, maternal DHA levels were negatively correlated with maternal and cord 17-HDHA levels. Best et al. observed a significant decrease in 7-HDHA levels at 34 vs. 14 weeks of pregnancy [24]. Our study supported this trend as 7-HDHA at delivery was lower in cord plasma compared to maternal plasma. Interestingly, we found that maternal 7-HDHA levels were negatively associated with infant birth weight percentile. Best et al. found an increased risk of spontaneous preterm birth with higher levels of 7-HDHA in mothers who received supplementation and had higher n-3 baseline levels. This association was not seen in women who received supplementation and had moderate to low n-3 status [24]. A synthetic 7-HDHA has been identified as a PPAR ligand, potentially highlighting a mechanistic pathway for 7-HDHA to influence infant growth [35].

## 4. Materials and Methods

### 4.1. Study Characteristics

The study’s eligibility for participation included mothers ≥19 years of age, delivering a live infant at Nebraska Medicine. All participating maternal–infant pairs were free of renal, metabolic, and hepatic diseases known to impair normal nutrient metabolism. Written informed consent was obtained from all subjects after admission to the labor and delivery unit. The UNMC’s Institutional Review Board approved the study.

### 4.2. Maternal Dietary Intake

All participants completed the Harvard Food Frequency Questionnaire (FFQ) that assesses dietary intake over the previous year. An advantage of FFQ compared to other nutrient intake assessments (e.g., 24 h recalls) is that this survey evaluates intake over time and can be used during pregnancy [36,37]. De-identified surveys were sent to the Harvard T. H. Chan School of Public Health for quantification based on previously established food nutrient content. Notably, the survey assessed supplement use and reported nutrient intake with and without supplementation. This tool has been validated in numerous adult populations, including pregnant women [36].

### 4.3. Maternal and Infant Outcomes

We used participants’ electronic medical records (EMRs) to extract relevant information and metrics, including maternal and infant outcomes, birth weight and length, head circumference, and associated percentiles (Table 11). Maternal smoking status and body mass index (BMI) were chosen as covariates due to previously reported associations between these parameters and inflammation, nutrient status, and infant outcomes [38,39].

### 4.4. Blood Sample Collection

Maternal and umbilical cord blood samples were collected by trained nurse personnel upon maternal admission to the hospital and at delivery. The research team obtained the samples within 12 h of collection, processed the specimens, and stored plasma fractions at −80 °C until metabolite analysis.

### 4.5. Quantification Methods for n-3 and n-6 FA Oxylipin

**Figure 3:**
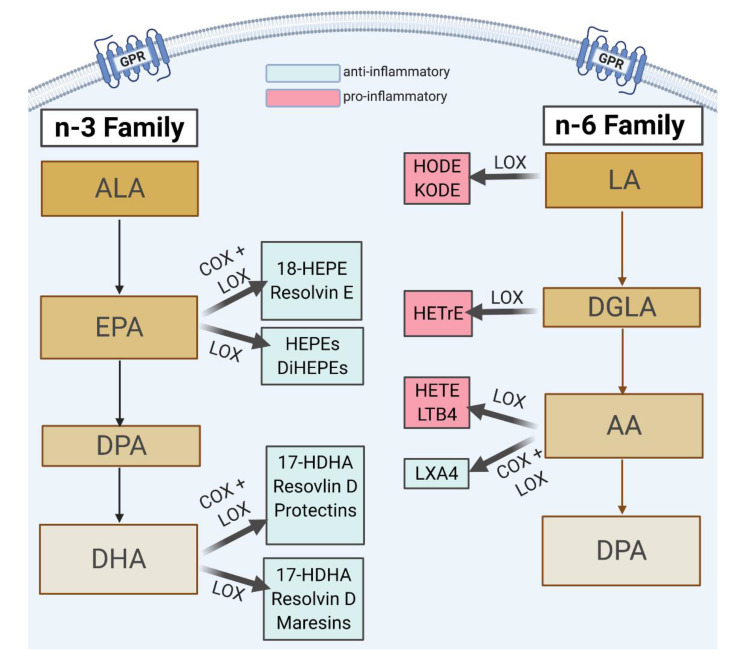
Demonstration of lipoxygenase enzymatic breakdown of metabolite with or without contributions from other pathways. Metabolites included in this study were formed through the lipoxygenase actions of 5-, 12-, and 15-LOX with or without modifications from other enzymes. This figure highlights metabolites that undergo breakdown by multiple enzymes. Created with BioRender.com (30 December 2021).

The levels of lipoxygenase pathway metabolites derived from linoleic acid (LA), dihomo-γ-linolenic acid (DGLA), arachidonic acid (AA), alpha-linolenic acid (ALA), eicosapentaenoic acid (EPA), and docosahexaenoic acid (DHA) were quantified in maternal and umbilical cord plasma using liquid chromatography–tandem mass spectrometric (LC–MS) approaches. Mediators assessed included those formed through lipoxygenase actions (including 5-, 12-, and 15-LOX) with or without additional modifications from other enzymes (e.g., COX metabolism)—Figure 3. Table 12 and Table 13 list the parent n-3 and n-6 FA nutrients and their metabolites included in the lipid panel.

#### 4.5.1. Fatty Acid Extraction

FAs were extracted as described by Yang et al. [40]. Waters Oasis-HLB cartridges (30 mg/30 μm) were treated with ethyl acetate (1 mL), methanol (2 × 1 mL), and 95:5 *v*/*v* water/methanol containing 0.1% acetic acid (1 mL). We mixed 100 μL of plasma with 7 μL of internal standard solution (stock concentration: 500 nM), 10 μL of butylated hydroxytoluene (BHT; stock concentration: 2 mg/mL), and 120 μL of H_2_O:methanol (MeOH) (95:5) containing 0.1% acetic acid. The resulting samples (240 μL) were then loaded onto pre-treated cartridges and washed twice with 750 μL of H_2_O/MeOH (95:5) containing 0.1% acetic acid. The aqueous plug was pulled from the cartridges using a high vacuum, and the cartridges were then dried further under a low vacuum for about 20 min. Waters Oasis-HLB cartridges were eluted into tubes with 250 μL of methanol followed by 1 mL of ethyl acetate into 2 mL tubes containing 6 μL of 30% glycerol in MeOH, a trap solution. The samples were dried under nitrogen and then dissolved in 70 μL of methanol containing 20 nM of 1-cyclohexyl-dodecanoic acid urea. The samples were then vortexed for 5 min, transferred to autosampler vials with low-volume inserts, and stored at −20 °C until further analysis.

#### 4.5.2. Liquid Chromatography–Mass Spectrometry

Samples (3 μL extracts) were analyzed by liquid chromatography coupled to electrospray ionization on a triple quadrupole mass spectrometer (Waters XEVO TQ-XS); the autosampler was cooled to 4 °C. Chromatographic separation was achieved on an Ascentis Express column (2.1 × 150 mm, 2.7 μm particles; Sigma-Aldrich Supelco, Darmstadt, Germany) at a flow rate of 0.35 mL/min at 40 °C using a gradient of 0.1% acetic acid and acetonitrile-isopropanol (90:10 *v*/*v*), as described [41]. All standards were dissolved in MeOH to achieve a final concentration of 1 μM. A standard preparation was used to build a 12-point calibration curve (97 pM–200 nM). Each point included 50 nM of deuterated internal standards. Individual calibration curves were generated by plotting oxylipin standard concentration vs. calculated response ratio (i.e., the ratio of oxylipin standard peak area and the corresponding internal standard peak area).

Data were processed using Skyline [42] software and Microsoft Excel. Calibration curves for each oxylipin were built using standards and deuterated internal standards. Calibration curves were calculated by linear regression with 1/x^2^ weighing. The calibration result was then corrected for dilution to determine the original oxylipin concentration in plasma. Results falling above or below the calibration curve were excluded from data analysis. With 60% or more of their samples above or below the standard curve, oxylipins were categorized as not detectable (ND). Appendix B includes the oxylipin library used and a detailed list of multiple reaction monitoring mode transitions. The chromatographs for the oxylipins are included in Appendix C.

### 4.6. Fatty Acid Quantification in Maternal Plasma

Gas chromatography with flame ionization detection (GC–FID) was used to analyze the plasma fatty acid composition at OmegaQuant Analytics LLC (Sioux Falls, SD, USA). FAs were extracted as described previously; in brief, plasma was mixed with boron trifluoride-methanol solution, vortexed, and heated in a hot bath at 100 °C [43]. Hexane and high-performance liquid chromatography-grade water was added after the solution was cooled. The tubes were centrifuged and the hexane layer was transferred to a GC vial. GC was performed using a GC-2010 Gas Chromatograph (Shimadzu Corporation, Columbia, MD, USA). A standard mixture of fatty acids (GLC OQ-A, NuCheck Prep, Elysian, MN, USA) was compared to the fatty acids of interest.

### 4.7. Statistics

For mothers who had twins, only information from the first twin was considered in our analysis. Descriptive statistics included means, standard deviations, medians, interquartile range (IQR), minimums and maximums for continuous data, and counts and percentages for categorical data. The Mann–Whitney U test was used to compare continuous measures between dichotomous groups. The Kruskal–Wallis test was used to compare measures between more than two groups. Fisher’s exact test was used to associate dichotomous categorical variables. Spearman correlation coefficients were used to assess relationships between continuous variables. The eicosanoids LTB4, 9-HEPE, and 8,15-DiHETE were detected in less than 40% of the samples. These metabolites were dichotomized to detectable vs. nondetectable to identify significant differences between the groups. McNemar’s test was used to assess potential associations between matching maternal-cord dichotomized metabolite values (i.e., ND vs. Determinate).

Linear regression modeling was performed on metabolites that correlated with birth weight, length, and head circumference at the *p* < 0.05 level in the univariate analysis. Associations of the metabolites with these birth measures were adjusted for obesity (>30 or ≤30 BMI (kg/m^2^)) and maternal smoking status (current/former vs. never) in the models. Metabolites were log-transformed in the regression analyses to meet the statistical assumptions of the models. *p* < 0.05 was considered statistically significant.

## 5. Conclusions

This study highlights the presence and potential influence of LOX pathway metabolites of both n-6 and n-3 FAs during pregnancy. Further, we demonstrate that LOX pathway metabolites potentially influence infant growth outcomes at delivery. Our study highlights the presence and potential influence of LOX pathway metabolites of both n-6 and n-3 PUFA on infant growth outcomes at delivery. Omega-6 and n-3 FAs have not been described in detail in maternal and cord plasma at the time of delivery. Therefore, this study adds to the breadth of metabolites described during pregnancy and related to infant growth. A limitation of this study is not identifying the direct causation of these eicosanoids on infant growth. Further studies are needed to understand the change in eicosanoids across pregnancy and their role in fetal development.

## Figures and Tables

**Table 1:** Maternal dietary intakes.

Maternal Daily Intake of FAs		
FAs	Median (g/Day) [IQR]	*n*
AA	0.15 [0.11]	103
LA	13.06 [7.91]	103
ALA	1.42 [0.75]	103
Gamma (γ)-LA	0.010 [0.01]	103
EPA	0.020 [0.06]	103
DPA	0.020 [0.03]	103
DHA	0.090 [0.15]	103
Total Omega n-3 (EPA + DHA + ALA + supplementation)	1.72 [1.01]	103
Total n-6	14.46 [7.43]	103
Ratio of n-6:n-3	8.73 [1.79]	102

**Table 2:** Maternal characteristics.

Race	%	*n*
White	62.18	74
African American	19.33	23
Hispanic	5.88	7
Asian or Pacific islander	0.84	1
American Indian	0.84	1
Other/unknown	10.92	13
Obesity	**%**	*n*
<30 kg/m^2^	72.28	73
≥30 kg/m^2^	27.72	28
Delivery Mode	**%**	*n*
Caesarian Section (C/S)	25.42	30
Vaginal	74.58	88
Maternal Smoking Status	**%**	*n*
None	77.97	92
Current smoker (at delivery)	11.02	13
Former smoker	11.02	13
Fish Oil/DHA Supplementation	**%**	*n*
No	79.31	92
Yes	20.69	24

**Table 3:** Infant baseline characteristics.

	*n*	Mean (SD)	Median [IQR]
Gestational age, weeks	119	38.17 (3.29)	39.20 [2.00]
Birthweight, kilograms (kg)	118	3.21 (0.77)	3.35 [0.80]
Birthweight percentile (%)	115	56.58 (28.86)	60.77 [45.69]
Head circumference, centimeters (cm)	115	34.09 (2.29)	34.30 [2.60]
Head circumference percentile (%)	115	58.5 (32.26)	63.58 [57.03]
Birth length, cm	114	49.1 (8.06)	49.50 [2.80]
Birth length percentile (%)	113	52.46 (33.41)	55.90 [61.30]
Sex	*n*	(%)	
Female	53	44.54	
Male	66	55.46	

**Table 4:** Correlations between maternal plasma levels of omega-6 parent nutrients (μg/mL) and omega-6 maternal and umbilical cord metabolite levels.

Maternal Parent Nutrient	Metabolite	*n*	r_s_	*p*-Value
Maternal Blood				
LA	13-HODE	52	0.097	0.49
	9-HODE	54	−0.086	0.54
	13-KODE	47	−0.15	0.33
DGLA	15-HETrE	55	0.027	0.84
AA	5-HETE	51	−0.094	0.51
	8-HETE	54	−0.032	0.82
	9-HETE	54	−0.067	0.63
	11-HETE	54	−0.073	0.60
	12-HETE	55	−0.068	0.62
	15-HETE	43	−0.24	0.11
	Lipoxin A4	36	0.095	0.58
	5,15-DiHETE	19	−0.41	0.079
Cord Blood				
LA	13-HODE	56	0.02	0.83
	9-HODE	56	0.056	0.68
	13-KODE	51	−0.096	0.50
DGLA	15-HETrE	56	−0.19	0.16
AA	5-HETE	55	−0.15	0.27
	8-HETE	56	−0.24	0.074
	9-HETE	56	−0.16	0.25
	11-HETE	56	−0.23	0.083
	12-HETE	50	−0.072	0.62
	15-HETE	55	−0.29	0.030 *
	Lipoxin A4	47	−0.40	0.0060 *
	5,15-DiHETE	33	0.051	0.78

* *p* ≤ 0.05.

**Table 5:** Maternal and cord oxylipin concentrations and correlations for n-6 FAs.

Precursor	Metabolite	Source	Median (nM) [IQR]	Rho (r_s_)	*p*-Value
LA	9-HODE	Maternal	18.94 [19.57]	0.091	0.37
		Cord	9.83 [6.40]		
LA	13-HODE	Maternal	20.99 [14.56]	0.22	0.029 *
		Cord	12.87 [8.70]		
LA	13-KODE	Maternal	2.31 [2.50]	0.25	0.018 *
		Cord	1.44 [0.90]		
DGLA	15-HETrE	Maternal	0.72 [0.83]	0.31	0.0011 *
		Cord	1.40 [1.09]		
AA	5-HETE	Maternal	5.86 [9.51]	0.49	<0.001 *
		Cord	5.86 [5.30]		
AA	8-HETE	Maternal	1.33 [1.76]	0.42	<0.001 *
		Cord	2.08 [1.36]		
AA	9-HETE	Maternal	1.06 [1.89]	0.37	0.0001 *
		Cord	2.24 [1.53]		
AA	11-HETE	Maternal	1.81 [2.55]	0.41	<0.001 *
		Cord	2.42 [2.68]		
AA	12-HETE	Maternal	3.84 [6.59]	0.26	0.0089 *
		Cord	8.32 [16.43]		
AA	15-HETE	Maternal	3.95 [5.62]	0.65	<0.001 *
		Cord	5.27 [5.22]		
AA	5,15-DiHETE	Maternal	0.27 [1.31]	0.75	<0.001 *
		Cord	0.16 [0.26]		
AA	Lipoxin A4	Maternal	10.29 [11.73]	0.12	0.31
		Cord	7.67 [7.61]		

* *p* < 0.05.

**Table 6:** Correlations between maternal plasma levels of parent nutrients (μg/mL) and maternal and umbilical cord metabolite levels.

Maternal Parent Nutrient	Metabolite	*n*	r_s_	*p*-Value
Maternal Blood				
ALA	9-HOTrE	56	0.011	0.94
EPA	5-HEPE	55	0.26	0.06
	9-HEPE	−	−	--
	12-HEPE	44	0.036	0.81
	15-HEPE	28	−0.22	0.27
DHA	7-HDHA	25	−0.28	0.18
	17-HDHA	39	−0.34	0.035 *
Cord Blood				
ALA	9-HOTrE	55	0.25	0.25
EPA	5-HEPE	56	0.11	0.41
	9-HEPE	−	**−**	**-**
	12-HEPE	49	−0.057	0.70
	15-HEPE	24	0.29	0.18
DHA	7-HDHA	44	−0.0020	0.99
	17-HDHA	46	−0.34	0.023 *

* *p* ≤ 0.05.

**Table 7:** Maternal and cord oxylipin concentrations and correlations for n-3 FAs.

Precursor	Metabolite	Source	Median (nM) [IQR]	r_s_	*p*-Value
ALA	9-HOTrE	Maternal	1.46 [1.60]	0.19	0.065
		Cord	0.47 [0.31]		
EPA	5-HEPE	Maternal	0.40 [0.61]	0.48	<0.001 *
		Cord	0.39 [0.29]		
EPA	9-HEPE	Maternal	0.19 [0.48]	0.36	0.080
		Cord	0.12 [0.06]		
EPA	12-HEPE	Maternal	0.34 [0.82]	0.0046	0.97
		Cord	0.25 [0.91]		
EPA	15-HEPE	Maternal	0.29 [0.27]	0.14	0.40
		Cord	0.20 [0.14]		
DHA	17-HDHA	Maternal	1.83 [2.63]	0.53	<0.001 *
		Cord	2.16 [1.94]		
DHA	7-HDHA	Maternal	1.36 [2.54]	0.56	<0.001 *
		Cord	1.16 [0.93]		

* *p* < 0.05.

**Table 8:** Correlations between dietary intakes and metabolite plasma levels.

Intake	Metabolite	r_s_	*p*-Value
n-6: n-3 Ratio	Maternal 15-HETE	−0.24	0.03
	Maternal 17-HDHA	−0.25	0.03
Total n-6	Cord 15-HETE	0.26	0.01
DHA	Maternal 9-HEPE	0.36	0.033
	Maternal 7-HDHA	0.36	0.003
	Maternal 17-HDHA	0.28	0.015
Total n-3	Cord 17-HDHA	0.22	0.04

Nonsignificant findings were not included in this table.

**Table 9:** Significant correlations between n-6 plasma metabolite levels and infant growth metrics.

Growth Metric	Metabolite	r_s_	*p*-Value
Birth Weight Percentile	Cord 9-HETE	−0.311	0.0012
	Maternal 12-HETE	−0.20	0.040
	Maternal 15-HETE	−0.21	0.043
	Maternal 5,15-DiHETE	−0.34	0.016
Birth Length Percentile	Cord 11-HETE	0.22	0.028
	Maternal 15-HETE	−0.21	0.046
	Cord 9-HODE	0.21	0.029
	Cord 13-HODE	0.22	0.026
	Cord 13-KODE	0.21	0.045
Birth Head Circumference Percentile	Cord 9-HETE	−0.22	0.023
Linear Regression			
Growth Metric	Metabolite	Beta (β)	*p*-Value
Birth Length Percentile	Cord 9-HODE	0.13	0.039
	Cord 13-HODE	0.20	0.0014
	Cord 13-KODE	0.19	0.016
Birth Weight Percentile	Maternal 5,15-DiHETE	−0.041	0.047

Nonsignificant findings were not included in this table.

**Table 10:** Significant relationships between omega-3 plasma metabolite levels and infant growth metrics.

Growth Metric	Metabolite	r_s_	*p*-Value
Birth Weight Percentile	Maternal 5-HEPE	−0.20	0.042
	Maternal 12-HEPE	−0.28	0.023
	Maternal 15-HEPE	−0.32	0.0089
	Maternal 7-HDHA	−0.34	0.0033
Birth Length Percentile	Cord 5-HEPE	0.21	0.036
Birth Head Circumference Percentile	Maternal 7 HDHA	−0.29	0.016
Linear Regression			
Growth Metrics	Metabolite	Beta (β)	*p*-Value
Birth Length Percentile	Cord 5-HEPE	0.12	0.025
Birth Weight Percentile	Maternal 7-HDHA	−0.062	0.025

Nonsignificant findings were not included in this table.

**Table 11:** Description of maternal and infant outcomes collected from the EMR.

Gestational Age	Measured in Weeks
Birth weight	Measured in grams immediately following birth
Birth length	Measured in centimeters immediately following birth
Head circumference	Measured in centimeters immediately following birth
Birth weight percentile	The WHO growth standards are used to calculate birth weight percentile. Out of 100 infants the same age, an infant in the 50th percentile will have 50 infants smaller and 50 infants larger than them
Birth length percentile	The WHO growth standards are used to calculate the birth length percentile. Out of 100 infants the same age, an infant in the 50th percentile will have 50 infants shorter and 50 infants longer than them
Head circumference percentile	The WHO growth standards are used to calculate the head circumference percentile. Out of 100 infants the same age, an infant in the 50th percentile for head circumference will have 50 infants with smaller heads and 50 infants with larger heads
Maternal Smoking Status	*Never smoker*: the mother never smoked
	*Current/Former smoker***:** the mother reported previously smoking or smoking at the time of delivery
BMI Categorization	*Not obese*: BMI ≤ 30 kg/m^2^
	*Obese*: BMI > 30 kg/m^2^
Delivery mode	Vaginal
	Caesarean

**Table 12:** n-3 FA metabolites and abbreviations.

Parent Nutrient	Metabolites	Abbreviation	Expected Effect
ALA	9-hydroxyoctadecatrenoic acid	9-HOTrE	Anti-inflammatory
EPA	5-hydroxyeicosapentaenoic acid	5-HEPE	
	12-hydroxyeicosapentaenoic acid	12-HEPE	
	15-hydroxyeicosapentaenoic acid	15-HEPE	
	18-hydroxyeicosapentaenoic acid	18-HEPE	
	Resolvin E1	RvE1	
DHA	17-hydroxydocosahexaenoic acid	17-HDHA	
	7-hydroxydocosahexaenoic acid	7-HDHA	
	Resolvin D1	RvD1	
	Resolvin D2	RvD2	
	Maresin 1	MaR1	

**Table 13:** n-6 FA metabolites and abbreviations.

Parent Nutrient	Metabolites	Abbreviation	Expected Effect
LA	9-hydroxy-octadecadienoic acid	9-HODE	Pro-inflammatory
	13-hydroxy-octadecadienoic acid	13-HODE	
	13-ketooctadecadienoic acid	13-KODE	
DGLA	15-hydroxyeicosatrienoic acid	15-HETrE	
AA	5-hydroxyeicosatetraenoic acid	5-HETE	
	8-hydroxyeicosatetraenoic acid	8-HETE	
	9-hydroxyeicosatetraenoic acid	9-HETE	
	11-hydroxyeicosatetraenoic acid	11-HETE	
	12-hydroxyeicosatetraenoic acid	12-HETE	
	15-hydroxyeicosatetraenoic acid	15-HETE	
	8,15-dihydroxyeicosatetraenoic acid	8,15-DiHETE	
	5,15-dihydroxyeicosatetraenoic acid	5,15-DiHETE	
	Lipoxin A4	LxA4	Anti-inflammatory
	Leukotriene B4	LTB4	Pro-inflammatory

## Data Availability

Not applicable.

## Appendix A

**Table A1:** Fatty acid concentrations in maternal plasma.

Parent Fatty Acid	Median	IQR
Maternal Linoleic (µg/mL)	1482.57	296.91
Maternal alpha-Linolenic (µg/mL)	35.64	10.90
Maternal Arachidonic (µg/mL)	272.70	109.78
Maternal Eicosapentaenoic (µg/mL)	5.96	5.26
Maternal Docosahexaenoic (µg/mL)	72.74	22.30

## Appendix B

The mass spectrometer was operated in multiple reaction monitoring (MRM) mode, and electrospray ionization was performed in negative ion mode. A detailed list of MRM transitions is presented in Table A2. Source and desolvation temperatures were 150 °C and 500 °C, respectively. Desolvation gas was set to 1000 L/h and cone gas to 150 L/h. Collision gas was set to 0.15 mL/min. All gases were nitrogen except the collision gas, which was argon. The capillary voltage was 2 kV in negative ion mode. Samples were analyzed in random order. A quality control sample was analyzed every eight injections to monitor system stability and performance. We achieved 100% recovery of the internal standards.

**Table A2:** Library of 52 targeted oxylipins with their corresponding retention time, product and metabolite *m*/*z*, and deuterated internal standard used for quantification.

Compound Name	RT (min)	Precursor Ion *m*/*z*	Product Ion *m*/*z*	Internal Standard
Leukotriene B4	11.01	335.2	195.1	Leukotriene B4-d4
(±) 7-HDHA	15.99	343.2273	201.2	15(S)-HETE-d8
(±) 7-HDHA	15.99	343.2273	141.1	15(S)-HETE-d8
(±) 17-HDHA	15.35	343.2	281.3	15(S)-HETE-d8
5-HETE	16.47	319.2	115.1	15(S)-HETE-d8
(±) 8-HETE	15.91	319.2	155.1	15(S)-HETE-d8
(±) 9-HETE	16.14	319.2	167.1	15(S)-HETE-d8
(±) 11-HETE	15.56	319.2	167.2	15(S)-HETE-d8
(±) 12-HETE	15.84	319.2	179.2	15(S)-HETE-d8
15-HETE	15.16	319.2	219.2	15(S)-HETE-d8
9(S)-HOTrE	13.14	293.2	171.1	9-HODE-d4
Lipoxin A4	8.03	351.2	115	Lipoxin A4-d5
Maresin 1	10.79	359.2	250	Maresin 1-d5
Maresin 1	10.79	359.2	221	Maresin 1-d5
Resolvin E1	4.88	349.2	195	Resolvin E1-d4
Resolvin D1	8.08	375.2	141	Resolvin D1-d5
Resolvin D1	8.08	375.2	121	Resolvin D1-d5
Resolvin D2	7.39	375.2	175	Resolvin D2-d5
Resolvin D2	7.39	375.2	233	Resolvin D2-d5
Lipoxin A4-d5	7.99	356.2	115	-
15(S)-HETE-d8	15.03	327.2	182	-
Maresin 1-d5	10.74	364.2	250	-
Resolvin E1-d4	4.86	353.2	197	-
Resolvin D1-d5	8.03	380.2	141	-
Resolvin D2-d5	7.35	380.2	175	-
Leukotriene B4-d4	10.96	339.2	197.1	-
Prostaglandin E2-d4	6.88	355.2	275.2	-
Thromboxane B2-d4	5.96	373.2	173	-
9-HODE-d4	14.74	299.2	172.1	-
9-HODE	14.65	295.2	171.1	9-HODE-d4
13-HODE	14.77	295.2	195.2	9-HODE-d4
5-HEPE	14.73	317.2	115.1	15(S)-HETE-d8
9-HEPE	14.35	317.2	149	15(S)-HETE-d8
12-HEPE	14.36	317.2	179.1	15(S)-HETE-d8
15-HEPE	13.97	317.2	219.2	15(S)-HETE-d8
5(S),15(S)-DiHETE	10.78	335.2	115.2	Leukotriene B4-d4
15(S)-HETrE	16.14	321.2	221.2	9-HODE-d4
13-KODE	15.26	293.2	113.1	9-HODE-d4
CUDA	11.31	339.2	214.3	-

## Appendix C

The chromatographs from the metabolites analyzed in this study are provided below.
